# Can Climatic Susceptibility Be Cured?

**Published:** 1898-01

**Authors:** B. Fincke


### CAN CLIMATIC SUSCEPTIBILITY BE CURED?

B. Fincke, M. D.

"The question, 'Can Climatic Susceptibility be Cured?' seems to me to admit of an affirmative answer, provided the cure comprise the truly homœopathic treatment by high potencies according to Hahnemann. He has already intimated (*Organon*, Section 244) that the climate in swampy regions does not even affect a healthy man in his earlier years with intermittent fever if he lives a proper life, free from want, fatigue, and destroying passions. The prevailing intermittents may at least only affect him after his arrival, but one or two of the finest doses of highly potentiated China-bark solution will soon free him from them. This means that his susceptibility to the climate where he lives will be cured, and this admits of its application in other climates. It is a common saying that Boston and Brooklyn have a bad climate for people liable to sore throats. This generalization may hold good for those whose throats have been not cured, but mismanaged, by allopathic and topical treatment, leaving the affection in a condition to be renewed at every unfavorable change in the weather—*e.g.*, to east winds and rain. These maltreated throats have given a bad name to Brooklyn; but my experience is to the effect that when they have been carefully and properly treated by high potential medication the climatic susceptibility will be cured, and people can live to a good old age without being hampered in this respect any more, as I have seen.

"There is also this to be considered, that men are able to live in any climate of the earth if they accommodate themselves to the requirements of it. And thus the climatic susceptibility is deadened or cured, and they are enabled to live anywhere, even to old age. *Per contra*, the climatic susceptibility enables sick people to be cured by its influence in transferring them from an injurious climate to a healthy one, as the change from the swamps to the mountains will cure without medication of any kind, as Hahnemann says."

Dr. Finch then moved that before discussing Dr. Fincke's paper, Dr. Carleton's paper be read. Carried.

Dr. Carleton then read the following paper, entitled:

